# The Cytochrome P450 Gene *CsCYP85A1* Is a Putative Candidate for *Super Compact-1* (*Scp-1*) Plant Architecture Mutation in Cucumber (*Cucumis sativus* L.)

**DOI:** 10.3389/fpls.2017.00266

**Published:** 2017-03-02

**Authors:** Hui Wang, Wanqing Li, Yaguang Qin, Yupeng Pan, Xiaofeng Wang, Yiqun Weng, Peng Chen, Yuhong Li

**Affiliations:** ^1^Horticulture College, Northwest A&F UniversityYangling, China; ^2^Horticulture Department, University of Wisconsin, MadisonWI, USA; ^3^Vegetable Crops Research Unit, United States Department of Agriculture–Agricultural Research Service, MadisonWI, USA; ^4^College of Life Science, Northwest A&F University, YanglingChina

**Keywords:** cucumber, plant architecture, dwarf, cytochrome P450, *CYP85A1*, BR biosynthesis

## Abstract

The dwarf or compact plant architecture is an important trait in plant breeding. A number of genes controlling plant height have been cloned and functionally characterized which often involve in biosynthesis or signaling of plant hormones such as brassinosteroids (BRs). No genes for plant height or vine length have been cloned in cucurbit crops (family *Cucurbitaceae*). From an EMS-induced mutagenesis population, we identified a *super compact* (SCP) mutant C257 which was extremely dwarf due to practically no internode elongation. Under dark growing condition, C257 did not undergo skotomorphogenesis and its mutant phenotype could be rescued with exogenous application of brassinolide (BL), suggesting SCP might be a BR-deficient mutant. Segregation analysis revealed a single recessive gene *scp-1* that was responsible for the SCP mutation. Map-based cloning combined with a modified MutMap identified *CsCYP85A1*, a member of the plant cytochrome P450 monooxygenase gene family, as the most possible candidate gene for *scp-1*, which encodes a BR-C6-oxidase in the BR biosynthesis pathway. We show that a SNP within the second exon of *scp-1* candidate gene caused the SCP phenotype. Three copies of the *CsCYP85A* gene are present in the cucumber genome, but only the *scp-1*/*CsCYP85A1* gene seemed active. The expression of *CsCYP85A1* was higher in flowers than in the leaves and stem; its expression in the wild type (WT) was feedback regulated by BL application. Its expression was reduced in C257 as compared with the WT. This was the first report of map-based cloning of a plant height gene in cucurbit crops. The research highlighted the combined use of linkage mapping, an improved MutMap method and allelic diversity analysis in natural populations in quick cloning of simply inherited genes in cucumber. The roles of *CsCYP85A1* in regulation of internode elongation in cucumber was discussed.

## Introduction

Dwarf plant mutations have played important roles in elucidating the regulatory molecular mechanisms of plant growth and development (Tanabe et al., 2005). Dwarfism in plants is also an important agronomic characteristic in crop breeding for lodging tolerance and increase of yield (Akter et al., 2015; Tamiru et al., 2015). Genes underlying dwarf mutations in a number of plant species have been cloned and functionally characterized, which in many cases are associated with biosynthesis and response pathways of plant hormones that regulate cell elongation and division (Clouse and Sasse, 1998; Richards et al., 2001; Sakamoto et al., 2004; Yamaguchi, 2008; Akter et al., 2015; Tamiru et al., 2015). For example, gibberellin (GAs) has been extensively investigated for its role in determining plant height. A remarkable example is the green revolution genes (*sd-1* in rice, and *Rht-B1b* and *Rht-D1b* in wheat for semi-dwarf varieties) that are involved in GA biosynthesis and signaling pathways (Peng et al., 1999; Spielmeyer et al., 2002; Hedden, 2003; Zeng et al., 2011).

Brassinosteroids (BRs), a class of polyhydroxylated plant steroidal hormones, have also been shown to play critical roles in the regulation of plant height (Tanabe et al., 2005; Li et al., 2013). Many studies have revealed that mutations of genes involved in BR biosynthesis and perception often display dwarfing phenotypes with significantly reduced stature, prolonged life span, delayed flowering and senescence, small dark-green and curled leaves, reduced male fertility and abnormal skotomorphogenic responses (Bishop et al., 1999; Mori et al., 2002; Fujioka and Yokota, 2003; Hong et al., 2003; Kwon et al., 2005; Li et al., 2013). However, from the plant breeding perspective, the loss-of-function mutations of these genes are not useful due to the extreme dwarf phenotypes; instead, mutants with moderate changes in BR levels by manipulating the expression of the BR biosynthesis or signaling genes may be more meaningful for crop improvement (Choe et al., 2001; Liu et al., 2007; Divi and Krishna, 2009).

The BR biosynthetic pathway and the genes involved in BR biosynthesis have been identified and characterized primarily in Arabidopsis, rice, and tomato (Choe, 2006; Nomura and Bishop, 2006; Kim et al., 2008; Divi and Krishna, 2009; Li et al., 2013). Brassinolide (BL), the most bioactive BR, is the end product in the BR biosynthetic pathway, which is synthesized from campesterol (CR), a C28 sterol and its 5α-reduction product campestanol through a grid like pathway (Choe, 2006; Nomura and Bishop, 2006). Most BR biosynthetic steps are catalyzed by cytochrome P450 monooxygenases.

In plants, cytochrome P450s (CYPs) form the third largest family of genes and the largest family of enzymes in plant metabolism, which represent around 1% of the plant protein-coding genes (Nelson and Werck-Reichhart, 2011). It has been estimated that there are 245 P450 genes in Arabidopsis, 270 in tomato, 334 in rice, 337 in soybean, 399 in potato, and 318 in maize (Schuler, 2015). However, relatively few P450 families and subfamilies existed in all plant species. The CYP85 clan that makes up ∼13% of all plant P450s is among the families that exist in all plant genomes surveyed so far, which includes *CYP85*, *CYP90*, and *CYP724B* subfamilies (Nomura and Bishop, 2006; Schuler, 2015). Many *CYP85* genes are involved in key steps in BR biosynthesis. Among them, *CYP85A* encodes BR-C6-oxidases catalyzing formation of BL and castasterone (CS), which are the two most active BRs (Castle et al., 2005; Nomura and Bishop, 2006; Cheon et al., 2013).

Despite their importance, biological functions of *CYP85A* genes remain unknown in most plant species. The *Dwarf* (*Dwarf/CYP85A1, CYP85A3*) gene in tomato was the first to be isolated and characterized (Bishop et al., 1996, 1999; Nomura et al., 2005). *CYP85A* genes coding for C-6 oxidase have also been characterized in Arabidopsis (*CYP85A1* and *CYP85A2*) (Salanoubat et al., 2000; Kim et al., 2005), pea (*CYP85A1* and *CYP85A6*) (Jager et al., 2007), rice (*BRD1/CYP85A1*) (Kim et al., 2008), maize (*CYP85A1*) (Makarevitch et al., 2012), and barley (*HvDWARF* and *HvBRD*) (Gruszka et al., 2016). These studies have revealed the critical roles of *CYP85A*-encoded C-6 oxidase in plant growth and organ development (Castle et al., 2005; Gruszka et al., 2016). Nevertheless, information on BR biosynthetic pathway is very limited in other plant species. Cloning and characterizing these key genes in BR biosynthesis is important to understand the regulatory mechanisms on plant growth and development by BR. Such knowledge is also important for genetic manipulation of plant architecture for crop improvement (Castle et al., 2005; Li et al., 2013).

Cucumber, *Cucumis sativus* L. (2*n* = 14) is an important vegetable crop worldwide. The ability to regulate plant height (vine length) is important in cucumber breeding to develop varieties to adapt to different production systems (Li Y. et al., 2011). Very few dwarf or compact mutants with reduced vine length (or plant height) have been described in cucumber including *compact* (Kauffman and Lower, 1976; Crienen et al., 2011; Li Y. et al., 2011), *compact-2* (Kubicki et al., 1986), and *super compac*t (Niemirowicz-Szczytt et al., 1996). Molecular markers linked with *cp* locus for the *compact* mutant have been identified (Li Y. et al., 2011), but no underlying genes have been cloned in any of these mutants. From an EMS mutagenesis population, we identified a super compact mutant C257. The objectives of the present study were to isolate and functionally characterize the gene, *scp-1*, that was responsible for the mutant phenotypes. We identified the *scp-1* candidate gene with the map-based cloning strategy, which was expedited with bulked segregant analysis and a modified MutMap method. We show that *scp-1* encodes a cucumber cytochrome P450 gene *CsCYP85A1* that played an important role in the BR biosynthesis pathway.

## Materials and Methods

### Plant Materials and Growth Conditions

The super compact mutant line C257 used in the present study was identified among 3038 M2 lines of an EMS (ethyl methanesulfonate)-induced mutagenesis population. The original donor line CCMC was a North China fresh market type inbred line with normal plant architecture and green leaf color C257 displayed an extremely dwarf phenotype with practically no internodes. This mutant was phenotypically similar to another EMS-induced cucumber ‘super compact’ (SCP hereinafter) mutant described by Niemirowicz-Szczytt et al. (1996) that had drastically reduced main stem length and no lateral branches, which was controlled by a single recessive gene *scp*. We designate our candidate gene as *scp-1* (hereinafter), it is possible that *scp* and *scp-1* could be two different genes, or different alleles of the same gene. However, we will never know this because the *scp* mutant reported by Niemirowicz-Szczytt et al. (1996) is not available to test and has presumably lost.

For genetic mapping and cloning of the mutant allele, two F_2_ mapping populations were developed from crosses of C257 with the parental line CCMC (578 plants) and the North American pickling cucumber line Gy14 (1356 plants). For genetic mapping of *scp-1*, F_2_-derived F_3_ families were also developed from 184 C257 × Gy14 F_2_ plants. For each F_3_ family, 36 plants were examined for segregation of plant height to infer F_2_ genotype at the *scp-1* locus. All plant materials for genetic mapping were grown in the greenhouses of Northwest A&F University under natural photoperiodic condition and were visually classified as either ‘super compact’ or normal (wild type, WT) throughout the development stage.

### Morphological Characterization and Photosynthesis-Related Parameters in SCP Mutant

We compared morphological traits between the mutant and WT under greenhouse conditions. Since seeds of homozygous C257 (*scp-1*) were not available, dormant and recessive F_2_ plants were used for phenotypic observations. Thirty mutant and WT plants were grown in the greenhouses of Northwest A&F University in three reps (10 per rep). At fruit setting stage, the vine length, width of the stem, number of nodes and internode length, leaf area were measured. We also examine the cells of stems in both genotypes with a scanning electronic microscope (SEM). Stem samples were fixed in 4% glutaraldehyde, rinsed with 0.1 M PBS for 10 min which was repeated four times. The samples were subsequently dehydrated in a graded ethanol series and substituted with 3-methylbutyl acetate. After drying with a HCP-2 critical point drier (Hitachi, Tokyo, Japan), the samples were dissected, sputter-coated with platinum and observed with a JSM-6360LV scanning electron microscope.

The mutant had very dark green leaves. We measured chlorophyll content and several photosynthetic parameters in mutant and WT plants. Leaf gas exchange was measured with the LI-6400XT Portable Photosynthesis System (LI-COR Biosciences, Lincoln, NE, USA) on the third functional leaves from the top at fruit setting stage under 1000 μmol m^-2^s^-1^ PPFD at a controlled CO_2_ supply (400 mmol CO_2_ mol^-1^ air). Additional parameters measured included: net photosynthetic rate (P_N_), stomatal conductance (g_s_), intercellular CO_2_ concentration (Ci), and transpiration rate (Tr). All measurements were performed in three replicates per genotype with five plants per rep. These leaf samples were also used for chlorophyll measurement with a Bio-Rad SmartSpec Plus spectrophotometer at 663, 645, and 470 nm, respectively, following Tang et al. (2004).

### Skotomorphogenesis of C257 Mutants and Response of the Mutant to BL Application

We examined possible link of the C257 mutation with the BR biosynthesis pathway. Homozygous dominant and recessive F_2_ plants at the *scp-1* locus from C257 × CCMC F_2_ plants were employed for investigation of skotomorphogenic responses in the dark. The seedling plants were kept in complete darkness at 30°C for 10 days. In a second experiment, these plants were kept in the growth chamber with 25–27°C day and 18–20°C night temperature and 16 h day/8 h dark photoperiod at *c.*150 μmol photons m^-2^ s^-1^. When the cotyledons were fully expanded, the seedlings were sprayed with BL of different concentrations at 2 days interval until flowering. Morphological data were collected 12 h after the last BL application.

### Molecular Mapping and Identification of Candidate Gene for *Scp-1*

The bulked segregation analysis (BSA) strategy was employed for quick identification of molecular markers linked with the *scp-1* locus. Two DNA bulks, the WT-bulk and the SCP-bulk were constructed by pooling equal amount of leaf tissue from five WT and five SCP mutant F_2_ plants of the C257 × Gy14 population, respectively. Polymorphic markers were identified by screening 2489 SSRs in the two parental lines and then applied to the two bulks. A framework map for *scp-1* was developed with polymorphic SSRs in 46 F_2_ plants. More closely linked markers were identified with the chromosome walking strategy following Li Y. et al. (2011). All newly developed markers were first screened for polymorphism between the two bulks, then the polymorphic ones were applied to the 46 F_2_ plants for linkage analysis. Very closely linked markers were applied to a larger F_2_ population with 184 individuals.

A modified MutMapping approach (Chen et al., 2014) was used for fine mapping and identification of candidate gene for *scp-1*. A mutant pool was constructed by pooling equal amount (100 mg) of leaf samples from 42 SCP mutant plants from the C257 × CCMC F_2_ population (578 plants). The bulked DNA of the mutant pool and the parental line CCMC were re-sequenced using an Illumina Hi-Seq 2000 sequencer (100 bp). The short reads of the mutant pool and CCMC were aligned against the cucumber reference genome 9930 (V2.0) (Li D.W. et al., 2011) using BWA software (Li and Durbin, 2009). SNP calling was conducted with SOAPsnp (Li et al., 2009). SNPs polymorphic between CCMC and the mutant pool were used for calculating SNP index. All SNPs were plotted onto the seven cucumber chromosomes. Candidate gene region was identified based on SNP index curve.

DNA extraction, PCR amplification of molecular markers and gel electrophoresis was conducted as described in Li Y. et al. (2011). Linkage analysis of the *scp-1* locus with molecular markers was performed with JoinMap 3.0. Initial linkage groups (chromosomes) were established at a LOD threshold of 4.0 and the Kosambi mapping function.

### Uniqueness Test of Causal SNP in Cucumber Natural Populations

MutMapping identified a causal SNP within the *scp-1* candidate gene. This SNP was converted into a dCAPS marker, P450-dCAPS, which was used to genotype the 184 F_2_ plants of C257 × Gy14. With this marker, we also conducted dCAPS assay among 412 lines of the natural cucumber population to examine if the SNP locus also exists in these lines following Pan et al. (2015). PCR amplicons were digested with the restriction enzyme *Dde* I followed by electrophoresis in 8% polyacrylamide gels to reveal the band patterns with silver staining. In the mutant, *Dde* I digestion cut the 254 bp strand into two bands of 19 and 235 bp; the WT only had one band of 254 bp in size.

### Cloning and Sequence Analysis of *Scp-1* Candidate Gene

Total RNA was extracted from leaves of the mutant and WT plants with Biozol total RNA Extraction Reagent (Bioer, China). First-strand cDNA was synthesized by Transcriptor First Strand cDNA Synthesis Kit (Roche, Germany). The cDNA sequence of *scp-1* was ligated to the linearized vector via homologous recombination using In-Fusion^TM^ Advantage PCR Cloning Kit (Clontech, USA). The ligation product was transformed into *E. coli* Top 10 using heat shock method. The positive clones were selected and sent for commercial sequencing with the Sanger method.

### Phylogenetic Analysis of Scp-1/CsCYP85A1 Homologs in Plants

We investigated the phylogenetic relationships of cucumber SCP-1 protein identified in the present study with 15 homologs from 11 plant species. The species, their protein sequence GenBank accession numbers in the NCBI database were as follows: *Pisum sativum* (pea) BAF56236.1; *Glycine soja* (soybean) KHN08349.1; *Morus notabilis* XP010099314.1; *Spinacia oleracea* (spinach) AMQ47459.1; *Solanum lycopersicum* (tomato) NP001234263.1 and NP001234520.1; *Nicotiana tabacum* (tobacco) ABG36709.1; *Arabidopsis thaliana* NP851105.1 and NP566852.1; *Zea mays* (maize) ACG46988; *Oryza sativa* (rice) XP015631644.1; *Hordeum vulgare* (barley) AEG64639.1. Multiple sequence alignment was performed with Clustal W in MEGA6.0^1^. A neighbor-joining tree (Saitou and Nei, 1987) was constructed based on 1,000 bootstrap replications, which was visualized using the Tree Explorer program in MEGA6.0.

### Expression Analysis of *Scp-1* Candidate Gene

We examined expression of the *scp-1* candidate gene, *CsCYP85A1*, and its two homologs in the cucumber genome (*C*s*CYP85A2* and *C*s*CYP85A3*) in WT and C257 mutant plants. Total RNA was isolated from various organs (root, stem, leaf, and flower) using Biozol total RNA Extraction Reagent (Bioer, China). First-strand cDNA was synthesized by 5x All-In-One RT MasterMix (ABM, Canada). The expression of candidate genes was analyzed by semi-quantitative RT-PCR and quantitative real-time PCR (qPCR). The cucumber *Ubiquitin extension protein* (*UBI-ep*) gene (Wan et al., 2010) was used as the internal reference. The primer pairs *CsCYP85A1*-F/*CsCYP85A1*-R and *CsCYP85A2*-F/Cs*CYP85A2*-R were used to measure the expression of *CsCYP85A1* and *CsCYP85A2*, respectively, in semi-quantitative RT-PCR. The qPCR was performed with the MyiQ^TM^ Single Color Real-Time PCR Detection System following manufacturer’s instructions. Gene expression level was calculated on the basis of the 2^-ΔΔ^*^Ct^* method (Livak and Schmittgen, 2001). Mean expression was based on three biological and three technical replications.

## Results

### Phenotypic and Photosynthetic Characterization of the Super Compact Mutant C257

**FIGURE 1:**
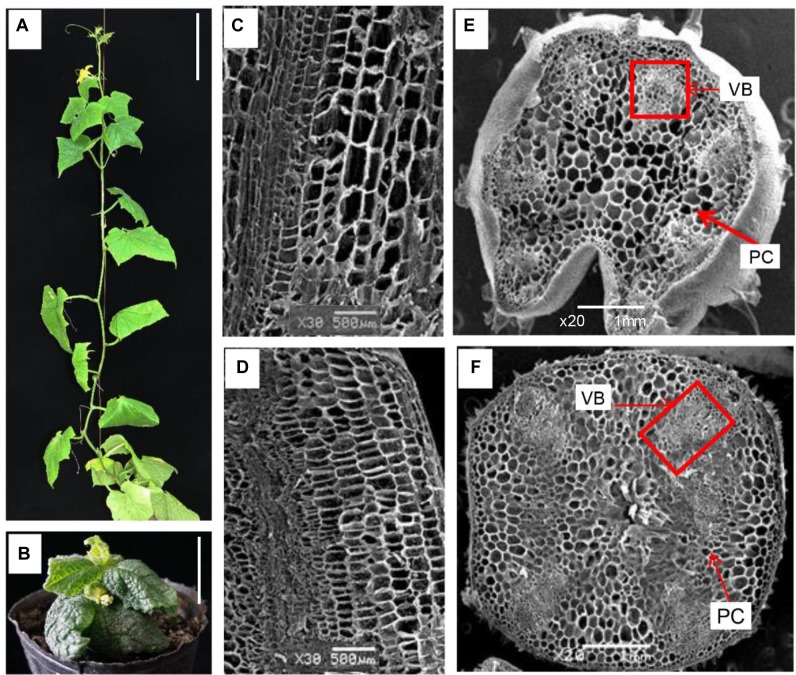
**Morphological characterization of WT (CCMC) and C257 mutant.** **(A,B)** Seventy-day-old plants of CCMC and C257, respectively. Longitudinal and cross sections of CCMC **(C,E)** and C257 **(D,F)** stems reveal shorter and wider cells of C257 than that of the WT. VB, vascular bundles; PC, parenchyma cells; Bar = 5 cm **(A,B)**; Bar = 500 μm **(C,D)**; Bar = 1 mm **(E,F)**.

A dwarf mutant C257 was discovered among 3,038 M2 lines derived from an EMS induced mutagenesis population from the CCMC parental line (WT). As compared with the WT, the mutant exhibited a super compact (SCP) phenotype (**Figure 1**). The internode length of C257 mutant was drastically reduced. The vine length of C257 adult plant was less than 5 cm (2.9% of WT), and the number of internodes was 37.5% of the WT with almost no elongation of the internodes (**Table 1**). The mutant plant had no tendrils; its leaves were more round shaped with wrinkled surface and dark green color (**Figure 1**). The mean of root length and volume of C257 was 48.9 and 22.4% of the WT, respectively (**Table 1**). Despite flower organs having its all principal parts, we failed to obtain selfed seeds from the mutant due to abnormal stigma and ovary.

**Table 1:** Morphological measurements of SCP mutant and WT CCMC at flowering stage.

Genotype	Tendril	Vine length (cm)	Internodes	Leaf length (cm)	Leaf area (cm^2^)	Root length (cm)	Root volume (cm^3^)	Fresh weight (g)
SCP (C257)	No	5.0 ± 0.2	6.6 ± 1.5	3.2 ± 0.1	9.5 ± 0.6	6.0 ± 0.3	1.5 ± 0.02	38.75 ± 1.54
WT (CCMC)	Yes	173.0 ± 4.3	17.0 ± 1.0	15.8 ± 0.5	235.4 ± 3.5	12.3 ± 0.2	6.7 ± 0.17	276.35 ± 10.42


Under a SEM, the cells of the stem in the mutant were shorter but wider than that of WT (**Figures 1C,D**) indicating defective cell elongation which might be the main reason for the reduced internode length. Examination of the cross sections of the stems revealed abnormal and reduced number of vascular bundles in C257, and the vascular bundles were randomly scattered among parenchyma cells with altered structure (**Figures 1E,F**) suggesting abnormal vascular patterning in the mutant. The defective vascular bundles seemed to be characteristic of BR mutants in plants (Hong et al., 2002; Ibanes et al., 2009; Li et al., 2013).

The leaves of the mutant exhibited dark green color. We measure chlorophyll contents and several photosynthetic parameters in the WT and C257 mutant. We did not find any significant differences in chlorophyll contents between the leaves of the mutant C257 and WT plants (**Table 2**), which suggesting that the dark green leaf color was not associated with higher pigment content. On the other hand, the net photosynthetic rate (P_N_), stomatal conductance (g_s_), and transpiration rate (Tr) of C257 were significantly decreased, but the intercellular CO_2_ concentration (Ci) was significantly increased in C257 as compared with the WT (**Table 2**), which may result in reduced photosynthetic activity in C257. Since the total chlorophyll contents were largely the same between C257 and WT, this may suggest that the decreased photosynthesis in C257 is not related to the chlorophyll and/or light absorption capacity.

**Table 2:** Measurements of photosynthetic parameters between C257 mutant and WT at flowering stage.

Genotype	Chlorophyll a	Chlorophyll b	Carotenoid	PN	gs	Ci	Tr
	(mg/g FW)	(mg/g FW)	(mg/g FW)	(μmolCO_2_⋅m^-2^s^-1^)	(mmol⋅m^-2^s^-1^)	(μmol⋅mol^-1^)	(mmol⋅m^-2^s^-1^)
SCP (C257)	1.46 ± 0.02a	0.52 ± 0.01	0.31 ± 0.01	9.97 ± 0.54^∗^	0.49 ± 0.02^∗^	340.23 ± 9.38^∗^	8.40 ± 0.86^∗^
WT (CCMC)	1.28 ± 0.02a	0.38 ± 0.02	0.28 ± 0.01	24.25 ± 1.25^∗^	0.75 ± 0.06^∗^	312.13 ± 10.28^∗^	9.07 ± 1.12^∗^


*The ^∗^means between C257 and CCMC are significantly difference at *P* < 0.05.*

### Skotomorphogenic Responses of C257 Mutant

**FIGURE 2:**
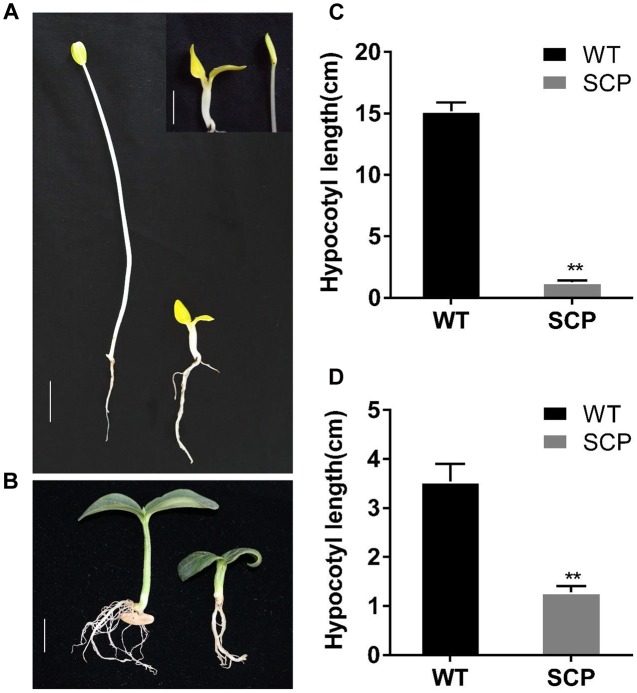
**Skotomorphogenic and photomorphogenic responses of WT and C257 SCP mutant.** The WT seedling exhibits typical skotomorphogenic (**A**, left, in dark) and photomorphogenic (**B**, left, under light) responses whereas C257 mutant has little hypocotyls elongation and open cotyledons in the dark (**A**, right). Hypocotyl length data in **(C, D)** were collected at 10 days after germination. ^∗∗^Indicate statistically significant differences of expression level based on Duncan’s test (*P* < 0.05). Bar = 1 cm.

The phenotypes observed in the C257 SCP mutant were very similar to BR-deficient and BR-insensitive mutants reported in Arabidopsis, rice, or tomato (Kwon and Choe, 2005). We suspected that C257 was deficient in BR biosynthesis or lacking of BR sensitivity. Previous work showed that BR-related dwarf mutants exhibited a de-etiolated phenotype with reduced hypocotyl elongation and open cotyledons when grown in the dark (Tanabe et al., 2005). We examined the growth behavior of C257 seeding grown in darkness. When grown in the dark, the WT seedling exhibited typical skotomorphogenic responses including excessive hypocotyl elongation and closured cotyledons; in contrast, the mutant seedling exhibited de-etiolation responses with absence of hypocotyl elongation and opened cotyledons (**Figures 2A,B**), which were similar to those observed in BR-related mutants. This may suggest possible involvement of BR signaling genes or biosynthesis genes in the mutation.

### Application of BL Can Partially Rescue Mutant Phenotypes in SCP Mutant

**FIGURE 3:**
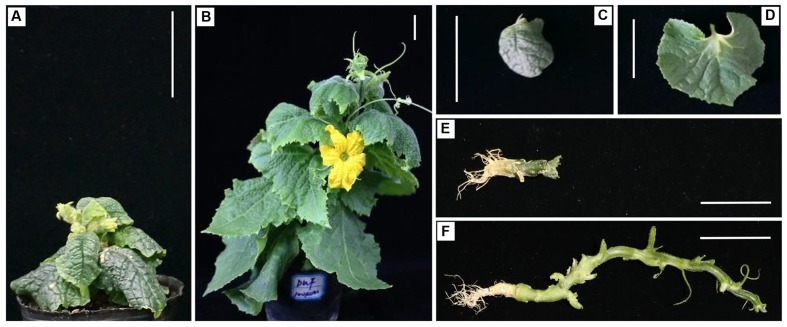
**Responses to BL application in C257 mutant.** At cotyledon stage, 100 μM BL was sprayed on C257 seedling plant. As compared with the control (water mock spray) **(A,C,E)**, BL application can partially rescue the mutant phenotype as seen from the plant height **(B)**, leaf growth **(D)**, and elongation of internodes **(F)**. Bar = 5 cm.

The skotomorphogenic responses of the mutant suggested that C257 is a possible a BR-deficient or BR-insensitive mutant. It has been reported that the dwarf phenotype of BR-deficient but not BR-insensitive mutants can be rescued by exogenously supplied BL (Castle et al., 2005). We investigated responses of C257 and the WT to BL application at five concentrations (0, 0.1, 1, 10, and 100 μM) and found that application of BL could partially rescue the mutant phenotypes in C257 (Supplementary Figures S1, S2). Application of BL increased plant height, petiole length, leaf area, internode length of the SCP mutant; the leaf color changed from dark green to light green although the total chlorophyll content remained unchanged; the BL-treated mutant also developed tendrils and normal female flowers (**Figure 3**). The effects of BL application were positively related with the BL concentrations. These observations suggested that C257 is a BR biosynthesis rather than a BR signaling mutant.

### Inheritance and Linkage Mapping of *Scp-1* Locus

To study the inheritance of SCP mutation in C257, we collected phenotypic data in two segregation populations derived from crosses of C257 with Gy14 and CCMC. All F_1_ plants had normal vine length indicating the recessive nature of the SCP mutation. Among 578 C257 × WT F_2_ plants, there were 448 normal and 130 SCP plants which was consistent with 3 to 1 segregation ratio (*P* = 0.16 in χ^2^ test against 3:1). Of the 1356 C257 × Gy14 F_2_ plants, 1038 had normal vine length and 318 exhibited SCP type (*P* = 0.18 in χ^2^ test against 3:1). These results confirmed that the super compact trait was controlled by a recessive nuclear gene, *scp-1*.

**FIGURE 4:**
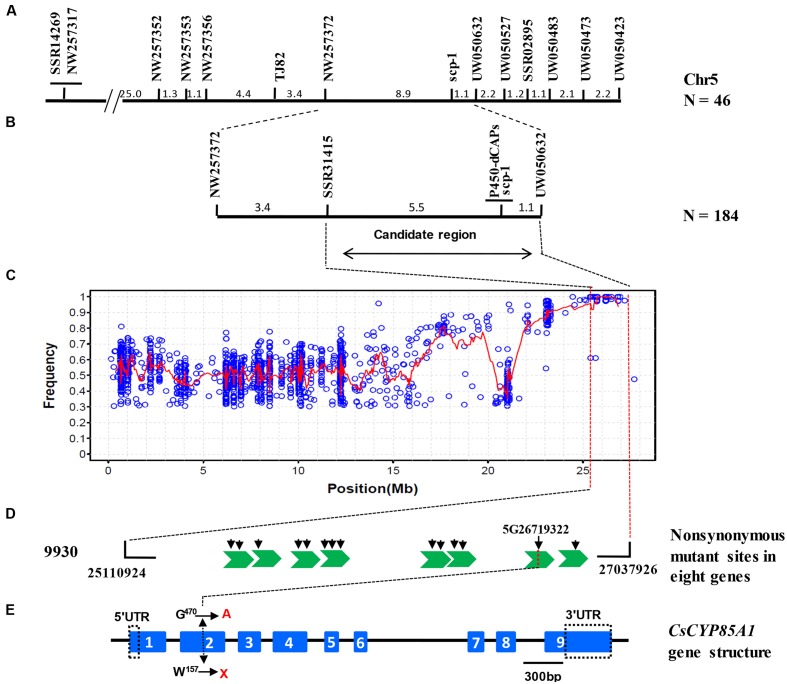
**Map-based cloning of *scp-1* locus.** Initial framework mapping with 46 F_2_ plants identified 11 SSR markers linked with *scp-1* in cucumber Chromosome 5 **(A)**. Linkage analysis with new markers in 184 F2 plants delimits the *scp-1* locus between SSR31415 and UW050632 **(B)**. SNP index plot shows possibly harboring causal mutations in chromosome 5 for C257 using MutMap. Red regression lines were obtained by averaging SNP indices from a moving window of five consecutive SNPs and shifting the window one SNP at a time. The *x*-axis value of each averaged SNP index was set at a midpoint between the first and fifth SNP **(C)**. Fourteen SNPs located in eight gene exon region were extracted. Green swallowtails and black arrows indicate genes and mutation sites, respectively **(D)**. The candidate gene encodes a putative cytochrome P450 protein *CsCYP85A1* which has nine exons and eight introns. The SNP in the second exon results in a tryptophan codon at 157 position (TGG) substitution to a stop codon (TAG). Blue rectangles with consecutive numbers and black line indicate exons and introns, respectively. Mutation identified in C257 are indicated **(E)**.

For linkage mapping, 2489 SSRs from seven cucumber chromosomes were screened for polymorphism between C257 and Gy14, of which 283 (11.4%) were polymorphic. Of the 283 markers, only two (SSR31415 and UW050483) were polymorphic between the WT-bulk and SCP-bulk. Both were located on cucumber chromosome 5. Linkage analysis of the two markers with 46 C257 × Gy14 F_2_ plants indicated that both were linked with the *scp-1* locus but were at the same side of *scp-1*. Based on the Gy14 draft genome sequences in this region, 205 additional SSR markers were tested and 15 polymorphic ones were identified. Eventually, 11 SSR were mapped with the closest markers being NW257372 and UW050632, which were 8.9 and 1.1 cm away from the *scp-1* locus, respectively (**Figure 4A**). In the region defined by the two flanking markers, 80 more SSRs were tested, but only one was mapped with 184 C257 × Gy14 F_2_ plants. Now, the *scp-1* locus was located between SSR31415 and UW050632, which were 5.5 and 1.1 cm away from the *scp-1* locus, respectively (**Figure 4B**). Detailed information of all markers placed on the map is provided in Supplementary Table S1.

### BSA and an Improved MutMap Approach Allowed Identification of *Scp-1* Candidate Gene

Since additional efforts to identify polymorphic SSRs in the target region did not yield any results, we employed an improved MutMap approach (Chen et al., 2014) to identify more polymorphisms in the target region. The CCMC parental line and a mutant bulk consisting of 42 homozygous recessive mutant F_2_ plants from the C257 × CCMC cross were re-sequenced using an Illumina Hi-Seq2000 sequencer. We obtained 102 million and 119 million reads for the mutant pool and WT, respectively. These reads were aligned to the 9930 draft genome (V2.0). For the mutant pool, 97.2% reads were mapped to the reference genome with 59.8% reads having unique alignments. For the WT, 97.1% reads had alignments with the reference genome. The alignment resulted in 111,490 SNPs between the mutant pool and WT. The estimated cucumber genome size was 367 Mbp implying a mutation frequency of 0.03% (or 0.3 mutation per kb DNA).

Among SNPs located within the target region defined by SSR31415 and UW050632 (25110924 to 27037926) (**Figure 4B**), we selected a subset SNPs meeting the following criteria: non-synonymous mutation; homozygous and SNP index = 1 (**Figures 4C,D**). This screening resulted in 14 SNPs that were located in exonic regions of eight genes (Supplementary Table S2).

We compared allelic variations of the 14 SNPs between 20 re-sequenced cucumber lines with normal vine length and the mutant pool with SCP phenotype. Of the 14 SNPs, the allelic variation for only one SNP, 5G26719322, was consistent with the phenotypes (Supplementary Table S2) suggesting it might be the causal mutation for the SCP phenotype. To examine the linkage relationship of this SNP with the *scp-1* locus, a dCAPS marker, P450-dCAPS, was developed from this SNP (primer information in Supplementary Table S1), which was used to genotype 184 F_2_ plants from C257 and Gy14 cross. This dCAPS marker was shown to co-segregate with *scp-1* locus (see Supplementary Figure S3A for example). Using this dCAPs marker, we also genotyped 412 cucumber lines in the natural populations with growth habit. All these lines shared the normal allele with the CCMC parental line (Supplementary Figure S3B) further confirming this SNP that was the causative nucleotide variation for the SCP phenotype in C257.

### Cloning and Sequence Analysis of *Scp-1* Candidate Gene

Genetic mapping and SNP uniqueness analysis supported the eighth gene delimited by SSR31415 and UW050632 (**Figure 4D**) as the candidate for the *scp-1* locus. Gene annotation predicted it to encode a cytochrome P450 protein CYP85A with 463 amino acid residues. We cloned this candidate gene and its cDNA from both C257 and the WT (primer sequences are provided in Supplementary Table S3). Alignment of the cDNA sequences and deduced amino acid sequences between the mutant and WT are presented in Supplementary files 1 and 2, respectively. The full-length coding sequence of this *CYP85A* gene was 1392b pin both C257 and the WT. There was only one SNP (G in WT to A in C257) at 470 bp site in the cDNA between C257 and WT, which was located in the second exon of the *scp-1* candidate gene that resulted in a tryptophan codon (TGG) to a stop codon (TAG) in mutant (**Figure 4E** and Supplementary file 2).

The cytochrome P450 constitutes a very large family with many members in the plant genome (Schuler, 2015). We conducted BLAST search in the 9930 and Gy14 cucumber draft genome assemblies using the *scp-1* candidate gene, designated as *CsCYP85A1* hereinafter (Nelson et al., 1996), as the query and identified two additional copies, *CsCYP85A2* and *CsCYP85A3* in the genome, which shared 84 and 83% identity with *CsCYP85A1*, respectively. Sequence alignment among the three homologs is shown in Supplementary files 1 and 2.

### Phylogenetic Relationships of Cucumber CsCYP85A1 with Its Homologs in Other Species

**FIGURE 5:**
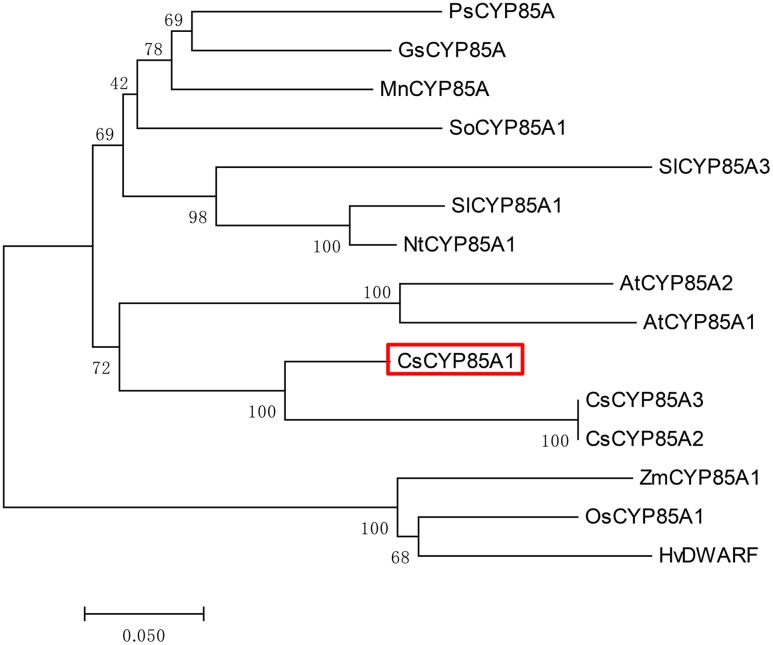
**Phylogenetic tree of CYP85A protein in cucumber and its homologs in 10 other species.** The phylogenetic tree was generated using the neighbor-joining method built in MEGA 7.0, and the inferred phylogeny was tested by bootstrap analysis with 1000 replicate datasets. Numbers shown at the tree forks indicate frequency of occurrence among all bootstrap iterations performed.

We examined the homologous sequences of cucumber SCP-1/CsCYP85A1 protein in other plants species including *Arabidopsis thaliana*, tomato, rice, maize, and soybean, and found this protein sequence was highly conserved with amino acid identity varying from 61 to 80% among those compared (Supplementary Table S4). To better understand the phylogenetic relationships of this gene among different species, we generated a phylogenetic tree based on the amino acid sequences of the CYP85A protein which is shown in **Figure 5**. The positions of different species in the Phylogram was consistently with their evolutionary relationships.

*CYP85A* has been shown to play important roles in the BR biosynthesis mediating the conversion of 6-deoxocastasterone (6-deoxoCS) to CS and CS to BL (Nomura et al., 2005). This sequence similarity among SCP-1 and its homologs suggested that that SCP-1 may play similar roles with the CYP85A in other species to catalyze reactions in BR biosynthesis.

### Expression Analyses of *Scp-1* (*CsCYP85A1*) in Various Tissues

We examined the transcript abundance of *CsCYP85A1*, *CsCYP85A2*, and *CsCYP85A3* with semi-quantitative RT-PCR in the root, leaf, male and female flowers as well as stem. The three genes shared high degree of DNA sequence homology. We tried to design PCR primers (Supplementary Table S3) to distinguish PCR products from each mRNA, but we could not separate the products from *CsCYP85A2* and *CsCYP85A3*. Fortunately, the expression of both *CsCYP85A2* and *CsCYP85A3* was almost undetectable whereas that of *scp-1/CsCYP85A1* was much higher in all tissues/organs of WT and the mutant (Supplementary Figure S4). This may suggest that *CsCYP85A2* and *CsCYP85A3* did not play important roles in cucumber growth and development, and their expression was not related with the super compact phenotype.

As compared with the WT, the expression of *scp-1/CsCYP85A1* in various organs/tissues was slightly lower (Supplementary Figure S4). This seemed to be inconsistent with results in other BR biosynthesis mutants with a truncated CYP85 protein (e.g., *CYP95A1* mutant *zmbrd1-m1* in maize and *CYP90D2/D2* mutant *d2-1* in rice) (Makarevitch et al., 2012; Li et al., 2013) in which the expression of *CYP85A* was not reduced in the mutant indicating *scp-1/CsCYP85A1* was different from other BR biosynthesis mutants.

### Feedback Regulation of CsCYP85A1 Expression by BL

**FIGURE 6:**
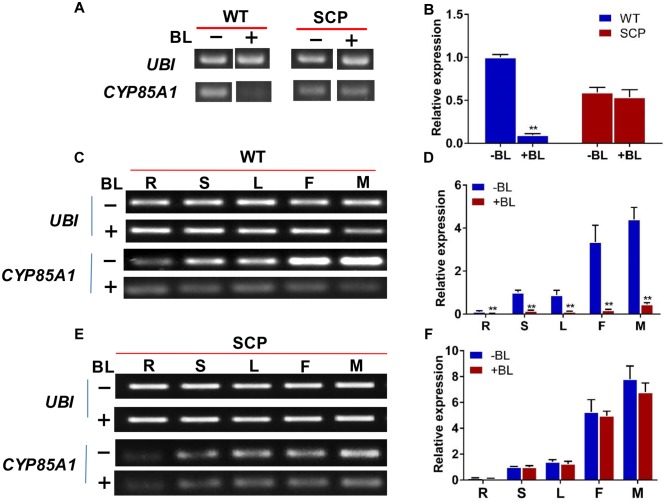
**Expression level of the *CsCYP85A1* candidate gene in WT and C257 plants with BL treatment revealed from semi-quantitative PCR** **(A,C,E)** and qPCR **(B,D,F)**. The expression of *scp-1/CsCYP85A1* in WT plants was dramatically reduced by treatment with 100 μM BL **(A,B)**, which was the same in various organs at flowering stage **(C,D)**. Exogenous BL treatment did not alter *scp-1/CsCYP85A1* expression in the mutant C257 **(A,B,E,F)**. ^∗∗^Indicates statistically significant differences of expression level based on *t*-test (*P* < 0.01). R, S, L, F, and M represents root, stem, leaf, female flower, and male flower, respectively.

Previous work indicated that transcription of the *CYP85A* gene is regulated in a feedback manner by BL, the end product of the BR biosynthesis pathway (e.g., Bancos et al., 2002; Hong et al., 2003). We investigated the expression of *CsCYP85A1* gene in both the mutant and WT after treatment with BL. Twelve hours after treatment with 100 μM BL of the seedlings, the expression of *scp-1*/*CsCYP85A1* was reduced dramatically in the WT but no change was observed in the mutant (**Figures 6A,B**) indicating that its expression was negatively feedback regulated by BL. Similar results were observed in different organs of the WT (**Figures 6C,D**) suggesting no organ specificity in feedback regulation by BL. The non-feedback regulation of the expression of *CsCYP85A1* in the mutant was also independent of different concentrations of BL (**Figures 6E,F** and Supplementary Figure S5).

## Discussion

### BSA and Modified MutMap Allows Rapid Identification of *Scp-1* Candidate Gene

In the present study, we reported map-based cloning of a SCP mutant allele *scp-1* in cucumber through BSA and a modified MutMap method. We first screened available SSR markers for polymorphisms between two parental lines (C257 and Gy14), and polymorphic markers were applied to the WT and SCP bulks, which allowed for quick location of the *scp-1* locus into a 1.09 Mbp region (**Figures 4A,B** and Supplementary Table S1). Since polymorphism in this region was relatively low, we failed to identify additional SSR makers. We took an improved MutMap approach by re-sequencing the recessive mutant DNA pool from the C257 × CCMC F_2_ plants as well as the CCMC parental line. This allowed us to identify 14 homozygous non-synonymous mutations (**Figure 4D**). Examination of allelic variations of the 14 SNPs among 20 re-sequenced cucumber lines revealed the only SNP that was completely consistent with the phenotype (SCP vs. normal vine type) (Supplementary Table S2). The dCAPs marker derived from this SNP was co-segregating with plant height in the segregating population. These data allowed to pin down the candidate gene for the *scp-1* locus to *CsCYP85A1*. Its candidacy was further confirmed by dCAPS assay with this marker among 412 cucumber lines in natural cucumber populations. Although additional evidence (for example, complementation test) may be needed to confirm the function of this gene in this mutant, this work exemplified integrated use of classic linkage analysis, BSA and next-generation high throughput sequencing, and genome-wide polymorphic loci analysis in quick identification and validation of simply inherited genes in cucumber.

MutMap took advantage of the efficiency of BSA and Illumina sequencing for quick mapping of target genes (e.g., Abe et al., 2012; Lun et al., 2016). In this method, the mutant pool DNA and the WT genome is sequenced. The sequencing reads from the WT genome is assembled and used as the reference for sequence alignment and SNP calling (Chen et al., 2014). In the present study, the reads of the mutant pool and CCMC WT were aligned directly to the 9930 reference genome. The resulting SNPs of mutant/9930 and WT/9930 were further compared to identify the causal SNP for the mutation. Our modified MutMap does not require assembled WT genome as reference, and thus is more cost-effective (Chen et al., 2014). However, while MutMap can reveal mutations in a mutant pool, it is difficult to identify the causal mutation(s). It was possible that the observed base variations indeed met the filtering criteria but were irrelevant to the phenotype in a particular mutant (Abe et al., 2012; Zhu et al., 2012; Chen et al., 2014). Therefore, combined use of linkage analysis in segregating populations and the modified MutMap method is preferred to ascertain the results.

### *CsCYP85A1* as the Candidate Gene for *Scp-1*

Map-based cloning identified *CsCYP85A1* as the most possible candidate gene for *scp-1*, and a single nucleotide change resulted in the super compact mutation in C257 mutant. This represents the first report to isolate and characterize a BR-deficient mutant in cucurbits (*Cucurbitaceae* family).

Brassinosteroids are steroidal phytohormones that are ubiquitously present in plants (Bishop and Yokota, 2001) and genes of the cytochrome P450 monooxygenase (P450) gene family play crucial roles in BR biosynthesis. The cucumber *CsCYP85A1* belongs to the CYP85 clan and CYP85A family. The plant *CYP85A* genes encode BR-6-oxidases that catalyze synthesis of two most biologically active forms of BRs (CS and BL) (Nomura and Bishop, 2006; Nelson and Werck-Reichhart, 2011). Mutations in different domains within this gene have been shown to be associate with extreme dwarf phenotypes in a number of plants. For example, the rice severe dwarf mutant *brd1-1* had 113 nucleotide deletion within the heme-binding site in the *CYP85A1/BRD1* gene (Mori et al., 2002). Another rice mutant *brd1-2* had a single nucleotide substitution at a site that is conserved among all members of CYP85, was severely dwarfed indicating the important of glycine residue for the expression of the BR-C6-oxidase activity (Hong et al., 2002). The *brd1-m1* mutant in maize, carried a premature stop codon after amino acid 165 that leads to lacking the whole CYP450 domain, has a severe dwarf phenotype (Makarevitch et al., 2012). Similar to *brd1-m1* in maize, the tomato extreme dwarf mutant *d(x)*, harbored a premature stop codon in exon 1, resulting in a truncated protein without CYP450 domain is also extreme dwarf phenotype (Bishop et al., 1996). In the present study, similar to the *Brd-1*/*CYP85A1* mutation in maize, the *CsCYP85A1*/*scp-1* mutation caused a stop codon after amino acid 157 which resulted in the deletion of the important functional domains of CYP450 including the heme-binding site (Supplementary file 2), which may render reduced function of the *scp-1* gene.

Consistent with their roles in BR biosynthesis, the *CYP85A* genes from different species shared high degree of homology. Among those species compared, the cucumber SCP-1 had the highest homology with *Arabidopsis thaliana* CYP85A (**Figure 5**). However, there are some differences in functions among these *CYP85A* genes. For example, two copies of the *CYP85A* gene, *CYP85A1* and *CYP85A2* are present in the Arabidopsis genome (Kwon et al., 2005). No abnormal phenotype was observed when a putative CYP85A homolog was disrupted (Shimada et al., 2003; Kwon and Choe, 2005), whereas the double mutant *cyp85a1*/*cyp85a2* displayed a severe dwarf phenotype suggesting function overlap between *CYP85A1* and *CYP85A2* in vegetative tissues (Kim et al., 2005; Kwon et al., 2005; Nomura et al., 2005). Similar to the Arabidopsis *CYP85A*, two *CYP85A* genes, *Dwarf*/*CYP85A1* and *CYP85A3*, exist in the tomato genome. *CYP85A1*/*DWARF* is expressed in vegetable tissues and fruits, while *CYP85A3* is preferentially expressed in developing fruits (Nomura et al., 2005). Consistent with their expression profiles, the extreme dwarf tomato mutant *d(x)* caused by the loss of function of the *Dwarf*/*CYP85A1* gene exhibits severe dwarfism and normal fruits (Nomura et al., 2005; Bishop et al., 2006). Both rice and maize contain only a single copy of functional homolog to *CYP85A1* (rice *OsBrd1*/*CYP85A1* and maize *ZmBrd1*/*CYP85A1*). Mutants defective in *OsBrd1*/*CYP85A1* or *ZmBrd1*/*CYP85A1* show severe growth retardation and sterility (Hong et al., 2002; Makarevitch et al., 2012; Gruszka et al., 2016), which may be due to that loss-of-function mutations are not compensated by any other paralogs. But the monocot barley has two genes encoding BR-6-oxidases, *HvDWARF* and *HvBRD* (Gruszka et al., 2016). The barley *brd1-d* mutant does not show any obvious reduction of fertility or leaf surface malformation implying that *HvDWARF* and *HvBRD* can be functionally partially compensated (Gruszka et al., 2016).

In this study, we identified three copies of *CYP85A* genes (*CsCYP85A1*, *CsCYP85A2*, and *CsCYP85A3*), but the expression of the second and third genes were almost undetectable (Supplementary Figure S4). The C257 mutant showed super compact phenotype with abnormal development of reproductive organs especially the female flowers. This suggested the functions of *CsCYP85A1* could not be compensated by other two homologs (*CsCYP85A2* and *CsCYP85A3*) indicating *CsCYP85A1* is a key gene in BR biosynthesis in the cucumber genome.

### Cucumber *Super Compact-1* Is a BR-Deficient Mutant

Previous studies in plants have shown that BR-deficient mutants have a distinctive dwarf phenotype such as dark green, rugose leaves, and a de-etiolated phenotype with reduced hypocotyl elongation and open cotyledons when grown in the dark (Tanabe et al., 2005). C257 was clearly also a BR-deficient mutant. This could be seen from the super compact or extreme dwarf appearance and the de-etiolated phenotype in the dark (**Figures 1**, **2**), which are also characteristics of BR-deficient mutants in other crops. In addition, the super compact phenotype of C257 mutants could be partially restored by the application of the BL (**Figure 3** and Supplementary Figures S1, S2). We show that the *scp-1* candidate gene encoded BR-C6-oxidase enzyme in BR biosynthesis pathway (**Figure 4** and Supplementary files 1 and 2), and the expression of *CsCYP85A1* was feedback regulated by exogenous BL application (**Figure 6**).

Exogenous application of BL could only partially restore the plant height of C257 mutant which also seemed to be BL dosage dependent (**Figure 3** and Supplementary Figure S1). The partial rescue of the mutant phenotype (plant height) has also been reported in BR-deficient mutants in rice and tomato. For example, BL treatment of tomato extreme dwarf mutant *d(x)* could restore the WT phenotypes, but the length of hypocotyl did not reach that of the WT tomato (Bishop et al., 1999). Bishop et al. (1999) hypothesized that the increased level of 6-DeoxoCS in the tomato *d(x)* mutant may act as a competitive inhibitor of the BR receptor and thus could prevent or reduce signaling for the downstream responses. Another reason may be the poor incorporation of the exogenously applied BL into mutant plants (Hong et al., 2002). It is possible that these reasons could also explain the partial recovery of vine length in C257 mutant after BL spray.

### Cucumber *Scp-1* Regulates Internode Elongation and Involves in Organ Development

Reduced plant height could be due to shortened internode length or fewer nodes or both. In C257 mutant, there was almost no internode elongation, and at the same time, the number of nodes was also reduced by 60% (**Figure 3** and **Table 1**). In WT cucumber, there is one leaf at each node. In C257 mutant, the number of leaves was more than that of nodes (Supplementary Table S5). Müssig (2005) found that BR cannot only promote internode elongation, but can also induce internode splitting. The reduced node numbers in the mutant was probably caused by reduced internode elongation. This could also be evidenced by the fact that no change of number of nodes was observed in the WT after application of BL (Supplementary Figure S2B). On the other hand, the application of BL on C257 mutant can restore the node numbers to the WT which was likely due to the elongation of the internodes which made the already formed nodes more visible. This was also observed in the rice *CYP90D* mutant (*d2-1*) (Li et al., 2013). Therefore, the extreme dwarf plant of C257 was due primarily to the inhibition of internode elongation caused by the mutation in most possible candidate gene *scp-1*/*CsCYP85A1*. The mutant also exhibited developmental defects in vascular bundles (**Figure 1**). These data suggest that *scp-1*/*CsCYP85A1* is required for elongation of cells in the stem as well as vascular differentiation and patterning.

## Author Contributions

HW performed the research and analyzed the data. WL participated in the experiments. YQ measured photosynthesis-related parameters. YP performed allelism test in cucumber natural population. XW examined the responses of the mutant to BL application. YW participated in data analysis and manuscript writing. PC and YL designed the experiments, analyzed the data and prepared a draft of the manuscript with inputs from all co-authors. All authors have read and approved the manuscript.

## Conflict of Interest Statement

The authors declare that the research was conducted in the absence of any commercial or financial relationships that could be construed as a potential conflict of interest.

## Footnotes

1 http://www.megasoftware.net/
