# Supplementary material for: The Cytochrome P450 Gene CsCYP85A1 Is a Putative Candidate for Super Compact-1 (Scp-1) Plant Architecture Mutation in Cucumber (Cucumis sativus L.)
Source: Front Plant Sci. 2017 Mar 2;8:266. doi: 10.3389/fpls.2017.00266 (PMC5332357; doi:10.3389/fpls.2017.00266)
Supplement: Supplementary file 2 [file Data_Sheet_1.DOCX]

**Supplemental file 1**

Alignment of cDNA sequence of *Scp-1*(*CsCYP85A1*) of C257 and 9930 cucumber lines as well as *CYP85A2* and *CYP85A3* from 9930. Nucleotide changes (SNPs) unique to C257 were highlighted in yellow. The causal SNP at position 471 results in premature termination of CYP85A1 protein synthesis.

**CLUSTAL 2.1 multiple sequence alignment**

scp-1 1 ATGGCCTTAGCCATGGTGATTATTGCTCTCTTTTTACTCCTCCTCTTTTGCTCCGCTTTA
CYP85A1 1 ATGGCCTTAGCCATGGTGATTATTGCTCTCTTTTTACTCCTCCTCTTTTGCTCCGCTTTA
CYP85A2 1 ATGGGCTTAGCCATGGTGATTGTTGGTCTGTTTTTACTCCTCTGTTTTTGCGCTGCTTTA
CYP85A3 1 ------------ATGGTGATTGTTGGTCTGTTTTTACTCCTCTGTTTTTGCGCTGCTTTA
consensus 1 ............*********.*** *** ************ ****** * ******

scp-1 61 CTCCGATGGAATGAAGTTCGATACAGCAAAAAAGGGTTGCCTCCCGGTACAATGGGATGG
CYP85A1 61 CTCCGATGGAATGAAGTTCGATACAGCAAAAAAGGGTTGCCTCCCGGTACAATGGGATGG
CYP85A2 61 CTCCGACGGAATGAAGTTCGATACATAAAAAATGGATTGCCTCCTGGTACAATGGGTTGG
CYP85A3 49 CTCCGACGGAATGAAGTTCGATACATAAAAAATGGATTGCCTCCTGGTACAATGGGTTGG
consensus 61 ****** ****************** ***** **.******** *********** ***

scp-1 121 CCTATTTTTGGTGAAACCACTGAGTTTCTTAAACAAGGTCCAAATTTTATGAAATCTCAA
CYP85A1 121 CCTATTTTTGGTGAAACCACTGAGTTTCTTAAACAAGGTCCAAATTTTATGAAATCTCAA
CYP85A2 121 CCTGTTTTTGGTGAAACCTCTGAGTTTCTTAAACAAGGTCCGAATTTTATGAATTCTAAA
CYP85A3 109 CCTGTTTTTGGTGAAACCTCTGAGTTTCTTAAACAAGGTCCGAATTTTATGAATTCTAAA
consensus 121 ***.************** **********************.*********** *** **

scp-1 181 AGAGCAAGGTATGGGAGCATTTTCAAGTCGCATATTTTGGGGTGTCCTACCATTGTTTCT
CYP85A1 181 AGAGCAAGGTATGGGAGCATTTTCAAGTCGCATATTTTGGGGTGTCCTACCATTGTTTCT
CYP85A2 181 AGAGCAAGGTATGGGAGCTTTTTCAAATCACATATTTTGGGATCTCCTACGGTTGTTTCT
CYP85A3 169 AGAGCAAGGTATGGGAGCTTTTTCAAATCACATATTTTGGGATCTCCTACGGTTGTTTCT
consensus 181 ****************** *******.**.***********.* ****** .********

scp-1 241 ATGGATCCAGAAGTGAATCGATATGTCTTAATGAATGAATCAAAAGGACTCGTTCCTGGT
CYP85A1 241 ATGGATCCAGAAGTGAATCGATATGTCTTAATGAATGAATCAAAAGGACTCGTTCCTGGT
CYP85A2 241 ATGGATCCAGAGGTTAATAGATATATTTTAATGAATGAATCTAAAGGACTCGTCGCTGGT
CYP85A3 229 ATGGATCCAGAGGTTAATAGATATATTTTAATGAATGAATCTAAAGGACTCGTCGCTGGT
consensus 241 ***********.** *** *****.* ************** *********** *****

scp-1 301 TACCCTCAATCCATGCTTGATATACTCGGAAAATGCAATATCGCCGCCGTACACGGCAGC
CYP85A1 301 TACCCTCAATCCATGCTTGATATACTCGGAAAATGCAATATCGCCGCCGTACACGGCAGC
CYP85A2 301 TATCCTCAATCTATGCTTGATATACTTGGAAAATGCAATATCGCTGCCGTACACGGCGAC
CYP85A3 289 TATCCTCAATCTATGCTTGATATACTTGGAAAATGCAATATCGCTGCCGTACACGGCGAC
consensus 301 ** ******** ************** ***************** ************..*

scp-1 361 GCTCATAAGCTTATGAGAGGTGCTCTGCTTGCTATTGTTAGTCCCACCATGATTAAAGAT
CYP85A1 361 GCTCATAAGCTTATGAGAGGTGCTCTGCTTGCTATTGTTAGTCCCACCATGATTAAAGAT
CYP85A2 361 GCTCATAAGCTTATGAGAGGTGCTCTACTTGCTATTGTTAGTCACACCATGATTAAAGAT
CYP85A3 349 GCTCATAAGCTTATGAGAGGTGCTCTACTTGCTATTGTTAGTCACACCATGATTAAAGAT
consensus 361 **************************.**************** ****************

scp-1 421 CGTCTTCTTCCTAACATTGATGAGTTTATGCGATCCCATTTGAGTAATT**A**GGATAATCAA
CYP85A1 421 CGTCTTCTTCCTAACATTGATGAGTTTATGCGATCCCATTTGAGTAATT**G**GGATAATCAA
CYP85A2 421 CGTCTTCTTCTTCAAGTTGATGAGTTTATGAGATCCCATTTGCGTAATT**G**GGATAGCCAA
CYP85A3 409 CGTCTTCTTCTTCAAGTTGATGAGTTTATGAGATCCCATTTGCGTAATT**G**GGATAGCCAA
consensus 421 ********** * * .************** *********** ********.*******. ***

scp-1 481 ATCATTGATATACAAGAAAAAACTAAAGAGATGGCTCTCCGCTCATCTATGAAGCAGATT
CYP85A1 481 ATCATTGATATACAAGAAAAAACTAAAGAGATGGCTCTCCGCTCATCTATGAAGCAGATT
CYP85A2 481 ATCATTGATATTCAAGAAAAAACTAAACAGATGGCTTTCTGGACATCTTTGAAGCAGACT
CYP85A3 469 ATCATTGATATTCAAGAAAAAACTAAACAGATGGCTTTCTGGACATCTTTGAAGCAGACT
consensus 481 *********** *************** ******** ** * ***** ********* *

scp-1 541 GCCGGATTCGAATCCGGTCCGTTATCGGAATCTTTCACCCCTGAGTTCTTTAAGTTAGTC
CYP85A1 541 GCCGGATTCGAATCCGGTCCGTTATCGGAATCTTTCACCCCTGAGTTCTTTAAGTTAGTC
CYP85A2 541 ACCAGTATCGAATCCGGTCCATTATGGGAATCTTTCACCATAGAATTCTTTAAGCTCGTC
CYP85A3 529 ACCAGTATCGAATCCGGTCCATTATGGGAATCTTTCACCATAGAATTCTTTAAGCTCGTC

consensus 541 .**.* *************.**** ************* **.********* * ***

scp-1 601 CTCGGCACTCTCTCTCTCCCTATCGATCTTCCCGGCACGAATTACCGCCGAGGAATTCAG
CYP85A1 601 CTCGGCACTCTCTCTCTCCCTATCGATCTTCCCGGCACGAATTACCGCCGAGGAATTCAG
CYP85A2 601 CTGGGCACTTTCTCGCTCCCAATCGATCTTCCAGGCACGAATTACCGTATAGGAGTTCAG
CYP85A3 589 CTGGGCACTTTCTCGCTCCCAATCGATCTTCCAGGCACGAATTACCGTATAGGAGTTCAG
consensus 601 ** ****** **** ***** *********** ************** ****.*****

scp-1 661 GCGAGGAAGAACATTATAAAGCTATTGGAACAGCTGATTAAAGAGAGAAGAGATTCAAAG
CYP85A1 661 GCGAGGAAGAACATTATAAAGCTATTGGAACAGCTGATTAAAGAGAGAAGAGATTCAAAG
CYP85A2 661 GCAAGGAATAACATAGTAAAGCTATTGAGGCAGCTGATTAATGAGAGAGGAAATTCAAAG
CYP85A3 649 GCAAGGAATAACATAGTAAAGCTATTGAGGCAGCTGATTAATGAGAGAGGAAATTCAAAG
consensus 661 **.***** ***** .***********...*********** ******.**.********

scp-1 721 ATTCAAAAAAATGACATGCTTGGTTATTTTATGAGTGAAGAAAACAAATATAAACTAAAT
CYP85A1 721 ATTCAAAAAAATGACATGCTTGGTTATTTTATGAGTGAAGAAAACAAATATAAACTAAAT
CYP85A2 721 ATTCAAAAAGATGACATGCTTGGATATTTATTGAGTGAAGAGAACAAATATAAACTAAAT
CYP85A3 709 ATTCAAAAAGATGACATGCTTGGATATTTATTGAGTGAAGAGAACAAATATAAACTAAAT
consensus 721 *********.************* ***** **********.******************

scp-1 781 GATGAAGAGATTATTGATCAAATCATTACTGTTTTGTATTCTGGCTATGAAACTGTTTCA
CYP85A1 781 GATGAAGAGATTATTGATCAAATCATTACTGTTTTGTATTCTGGCTATGAAACTGTTTCA
CYP85A2 781 GAGGAACAGATTATTGATCAAGTCATTACTGTTTTCTATTCTGGTTATGAAACTGTTTCA
CYP85A3 769 GAGGAACAGATTATTGATCAAGTCATTACTGTTTTCTATTCTGGTTATGAAACTGTTTCA
consensus 781 ** *** **************.************* ******** ***************

scp-1 841 ACAACTTCAATGATGGCTGTAAAGTTCCTCCATGATCATCCCAAAGTTCTTCAGCAACTA
CYP85A1 841 ACAACTTCAATGATGGCTGTAAAGTTCCTCCATGATCATCCCAAAGTTCTTCAGCAACTA
CYP85A2 841 ACAACTTCAATGATGGCTGTAAAGTTCCTTCATGATCATCCCAAAGTTCTTCAGCAACTC
CYP85A3 829 ACAACTTCAATGATGGCTGTAAAGTTCCTTCATGATCATCCCAAAGTTCTTCAGCAACTC
consensus 841 ***************************** *****************************

scp-1 901 AGAGAAGAACATTTAGCAATAAGAGAGAAGAAGAAAAATCCTGAGGACCCCATTGATTGG
CYP85A1 901 AGAGAAGAACATTTAGCAATAAGAGAGAAGAAGAAAAATCCTGAGGACCCCATTGATTGG
CYP85A2 901 AGAGAAGAACATTTAGCATTAAGAGAGAAGAA---AAATCCCAAGGACCCGATCGATTGG
CYP85A3 889 AGAGAAGAACATTTAGCATTAAGAGAGAAGAA---AAATCCCAAGGACCCGATCGATTGG
consensus 901 ****************** *************...****** .******* ** ******

scp-1 961 GATGATCTTAAAGCAATGAAATTCACTCGTGCGGTGATATTTGAAACATCAAGGTTGGCT
CYP85A1 961 GATGATCTTAAAGCAATGAAATTCACTCGTGCGGTGATATTTGAAACATCAAGGTTGGCT
CYP85A2 958 GATGATTTTAAAGCAATGGAGTTCACTCGTGCGGTAATTTTTGAAACATCAAGGCTAGCT
CYP85A3 946 GATGATTTTAAAGCAATGGAGTTCACTCGTGCGGTAATTTTTGAAACATCAAGGCTAGCT
consensus 961 ****** ***********.*.**************.** *************** *.***

scp-1 1021 ACTATTGTTAATGGGGTTTTGAGGAAAACTACAAAAGATATGGAATTAAATGGCTTTCTA
CYP85A1 1021 ACTATTGTTAATGGGGTTTTGAGGAAAACTACAAAAGATATGGAATTAAATGGCTTTCTA
CYP85A2 1018 ACTGTTGTTAATGGAGTTTTGAGGAAAACTACAAAGGATTTGGAAGTAAATGGGTTTCTA
CYP85A3 1006 ACTGTTGTTAATGGAGTTTTGAGGAAAACTACAAAGGATTTGGAAGTAAATGGGTTTCTA
consensus 1021 ***.**********.********************.*** ***** ******* ******

scp-1 1081 ATTCCAAAAGGATGGAGGATTTATGTGTATACAAGAGAGATCAACTATGACCCTTTTCTT
CYP85A1 1081 AT**T**CCAAAAGGATGGAGGATTTATGTGTATACAAGAGAGATCAACTATGACCCTTTTCTT
CYP85A2 1078 AT**T**CCCAAGGGATGGAGGATTTATGTGTATAATCGAGAGATAAACTATGACCCTTTTCTC
CYP85A3 1066 AT**T**CCCAAGGGATGGAGGATTTATGTGTATAATCGAGAGATAAACTATGACCCTTTTCTC
consensus 1081*******.** **.********************** ******* *****************

scp-1 1140 TATTCTGAACCATTCACCTTCAACCCTTGGAGATGGCTGGATAACAACTTAGAGTCCAAT
CYP85A1 1141 TATTCTGAACCATTCACCTTCAACCCTTGGAGATGGCTGGATAACAACTTAGAGTCCAAT
CYP85A2 1138 TATTCCGAACCATACACTTTCAACCCTTGGAGATGGCTGGACAAAAGCTTAGAGTCCCAT
CYP85A3 1126 TATTCCGAACCATACACTTTCAACCCTTGGAGATGGCTGGACAAAAGCTTAGAGTCCCAT
consensus 1141 ***** ******* *** *********************** ** *.********** **

scp-1 1200 AATTACTTCTTCATCTTTGGTGGTGGAACAAGACTTTGTCCTGGCAAAGAATTGGGAATT
CYP85A1 1201 AATTACTTCTTCATCTTTGGTGGTGGAACAAGACTTTGTCCTGGCAAAGAATTGGGAATT
CYP85A2 1198 AATTATTTTTTCCTCTTTGGCGGTGGAATTAGGCTTTGTCCTGGCAAAGAATTGGGAATT
CYP85A3 1186 AATTATTTTTTCCTCTTTGGCGGTGGAATTAGGCTTTGTCCTGGCAAAGAATTGGGAATT
consensus 1201 ***** ** *** ******* ******* **.***************************

scp-1 1260 GCTGAGATTTCTACTTTCTTACATTACTTTGTAACTAAATACAGATGGGAAGAAGTTGGA
CYP85A1 1261 GCTGAGATTTCTACTTTCTTACATTACTTTGTAACTAAATACAGATGGGAAGAAGTTGGA
CYP85A2 1258 GCAGAGGTTTCTACATTTTTACATTACTTTGTAACTAAATACAGGTGGGAAGAAGTTGGA
CYP85A3 1246 GCAGAGGTTTCTACATTTTTACATTACTTTGTAACTAAATACAGGTGGGAAGAAGTTGGA
consensus 1261 ** ***.******* ** **************************.***************

scp-1 1320 GGAGATAAGCTCTTGAAATTTCCAAGAGTGGAAGCACCAAGTGGGTTCCACATTAGGGTT
CYP85A1 1321 GGAGATAAGCTCTTGAAATTTCCAAGAGTGGAAGCACCAAGTGGGTTCCACATTAGGGTT
CYP85A2 1318 GAATGTAAGCTCTTGAAATTTCCAAGAGTTAAAGCTCGGAATGGGTTTCACATTAGGGTT
CYP85A3 1306 GAATGTAAGCTCTTGAAATTTCCAAGAGTTAAAGCTCGGAATGGGTTTCACATTAGGGTT
consensus 1321 *.* .************************ .**** * .*.****** ************

scp-1 1380 TCATCTTACTGA
CYP85A1 1381 TCATCTTACTGA
CYP85A2 1378 TCATCCTACTGA
CYP85A3 1366 TCATCCTACTGA
consensus 1381 ***** ******

**Supplemental file 2**

Alignment of SCP (CYP85A1) protein sequences of C257 and WT cucumber lines with CYP85A2 and CYP85A3 in WT. Conserved residues are marked by asterisks (*). Underlined amino acid residues indicate special domains. Various domain regions, such as the anchor region, the Pro-rich region and the heme binding domain, are labeled.

Membrane anchor region

re

Proline-rich

SCP 1 MALAMVIIALFLLLLFCSALLRWNEVRYSKKGLPPGTMGWPIFGETTEFLKQGPNFMKSQ

CYP85A1 1 MALAMVIIALFLLLLFCSALLRWNEVRYSKKGLPPGTMGWPIFGETTEFLKQGPNFMKSQ
CYP85A2 1 MGLAMVIVGLFLLLCFCAALLRRNEVRYIKNGLPPGTMGWPVFGETSEFLKQGPNFMNSK
CYP85A3 1 ----MVIVGLFLLLCFCAALLRRNEVRYIKNGLPPGTMGWPVFGETSEFLKQGPNFMNSK
consensus 1 ....***..***** ** **** ***** * **********.****.********** *

SCP 61 RARYGSIFKSHILGCPTIVSMDPEVNRYVLMNESKGLVPGYPQSMLDILGKCNIAAVHGS
CYP85A1 61 RARYGSIFKSHILGCPTIVSMDPEVNRYVLMNESKGLVPGYPQSMLDILGKCNIAAVHGS
CYP85A2 61 RARYGSFFKSHILGSPTVVSMDPEVNRYILMNESKGLVAGYPQSMLDILGKCNIAAVHGD
CYP85A3 57 RARYGSFFKSHILGSPTVVSMDPEVNRYILMNESKGLVAGYPQSMLDILGKCNIAAVHGD
consensus 61 ****** ******* **.**********.********* ********************

SCP 121 AHKLMRGALLAIVSPTMIKDRLLPNIDEFMRSHLSN--------------MALRSSMKQI
CYP85A1 121 AHKLMRGALLAIVSPTMIKDRLLPNIDEFMRSHLSNWDNQIIDIQEKTKEMALRSSMKQI
CYP85A2 121 AHKLMRGALLAIVSHTMIKDRLLLQVDEFMRSHLRNWDSQIIDIQEKTKQMAFWTSLKQT
CYP85A3 117 AHKLMRGALLAIVSHTMIKDRLLLQVDEFMRSHLRNWDSQIIDIQEKTKQMAFWTSLKQT
consensus 121 ************** ******** ..******** *..............** .*.**

SCP 167 AGFESGPLSESFTPEFFKLVLGTLSLPIDLPGTNYRRGIQARKNIIKLLEQLIKERRDSK
CYP85A1 181 AGFESGPLSESFTPEFFKLVLGTLSLPIDLPGTNYRRGIQARKNIIKLLEQLIKERRDSK
CYP85A2 181 TSIESGPLWESFTIEFFKLVLGTFSLPIDLPGTNYRIGVQARNNIVKLLRQLINERGNSK
CYP85A3 177 TSIESGPLWESFTIEFFKLVLGTFSLPIDLPGTNYRIGVQARNNIVKLLRQLINERGNSK
consensus 181 ***** **** ********* ************ *.*** **.*** *** ** **

SCP 227 IQKNDMLGYFMSEENKYKLNDEEIIDQIITVLYSGYETVSTTSMMAVKFLHDHPKVLQQL
CYP85A1 241 IQKNDMLGYFMSEENKYKLNDEEIIDQIITVLYSGYETVSTTSMMAVKFLHDHPKVLQQL
CYP85A2 241 IQKDDMLGYLLSEENKYKLNEEQIIDQVITVFYSGYETVSTTSMMAVKFLHDHPKVLQQL
CYP85A3 237 IQKDDMLGYLLSEENKYKLNEEQIIDQVITVFYSGYETVSTTSMMAVKFLHDHPKVLQQL
consensus 241 *** ***** .*********.* ****.*** ****************************

Heme-binding domain

SCP 287 REEHLAIREKKKNPEDPIDWDDLKAMKFTRAVIFETSRLATIVNGVLRKTTKDMELNGFL
CYP85A1 301 REEHLAIREKKKNPEDPIDWDDLKAMKFTRAVIFETSRLATIVNGVLRKTTKDMELNGFL
CYP85A2 301 REEHLALREKK-NPKDPIDWDDFKAMEFTRAVIFETSRLATVVNGVLRKTTKDLEVNGFL
CYP85A3 297 REEHLALREKK-NPKDPIDWDDFKAMEFTRAVIFETSRLATVVNGVLRKTTKDLEVNGFL
consensus 301 ******.****.** ******* *** **************.***********.*.****

SCP 347 IPKGWRIYVYTREINYDPFLYSEPFTFNPWRWLDNNLESNNYFFIFGGGTRLCPGKELGI
CYP85A1 361 IPKGWRIYVYTREINYDPFLYSEPFTFNPWRWLDNNLESNNYFFIFGGGTRLCPGKELGI
CYP85A2 360 IPKGWRIYVYNREINYDPFLYSEPYTFNPWRWLDKSLESHNYFFLFGGGIRLCPGKELGI
CYP85A3 356 IPKGWRIYVYNREINYDPFLYSEPYTFNPWRWLDKSLESHNYFFLFGGGIRLCPGKELGI
consensus 361 ********** *************.********* *** ****.**** **********

SCP 407 AEISTFLHYFVTKYRWEEVGGDKLLKFPRVEAPSGFHIRVSSY
CYP85A1 421 AEISTFLHYFVTKYRWEEVGGDKLLKFPRVEAPSGFHIRVSSY
CYP85A2 420 AEVSTFLHYFVTKYRWEEVGECKLLKFPRVKARNGFHIRVSSY
CYP85A3 416 AEVSTFLHYFVTKYRWEEVGECKLLKFPRVKARNGFHIRVSSY
consensus 421 **.***************** ******** * *********
