# Supplementary figures and images for: The Cytochrome P450 Gene CsCYP85A1 Is a Putative Candidate for Super Compact-1 (Scp-1) Plant Architecture Mutation in Cucumber (Cucumis sativus L.)
Source: Front Plant Sci. 2017 Mar 2;8:266. doi: 10.3389/fpls.2017.00266 (PMC5332357; doi:10.3389/fpls.2017.00266)

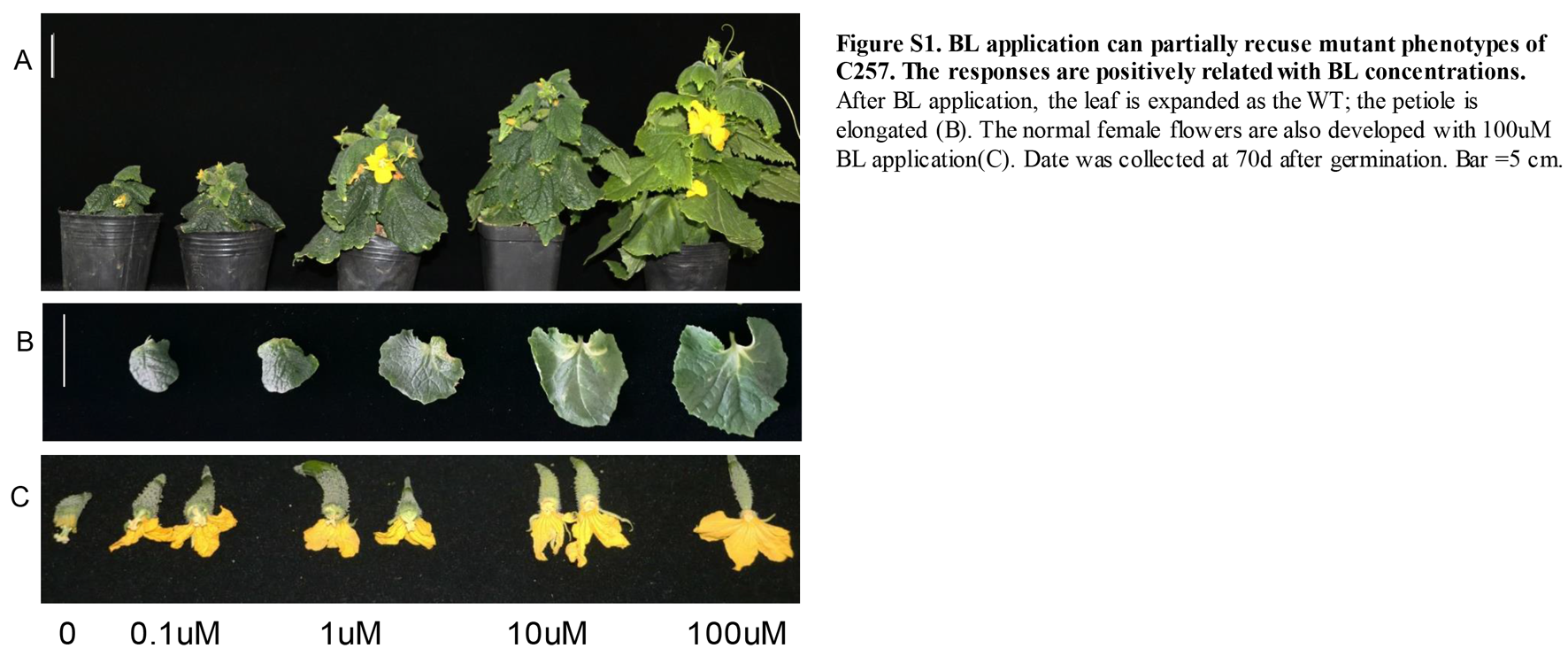

Supplement: Supplementary file 3 [file Image_1.TIF]

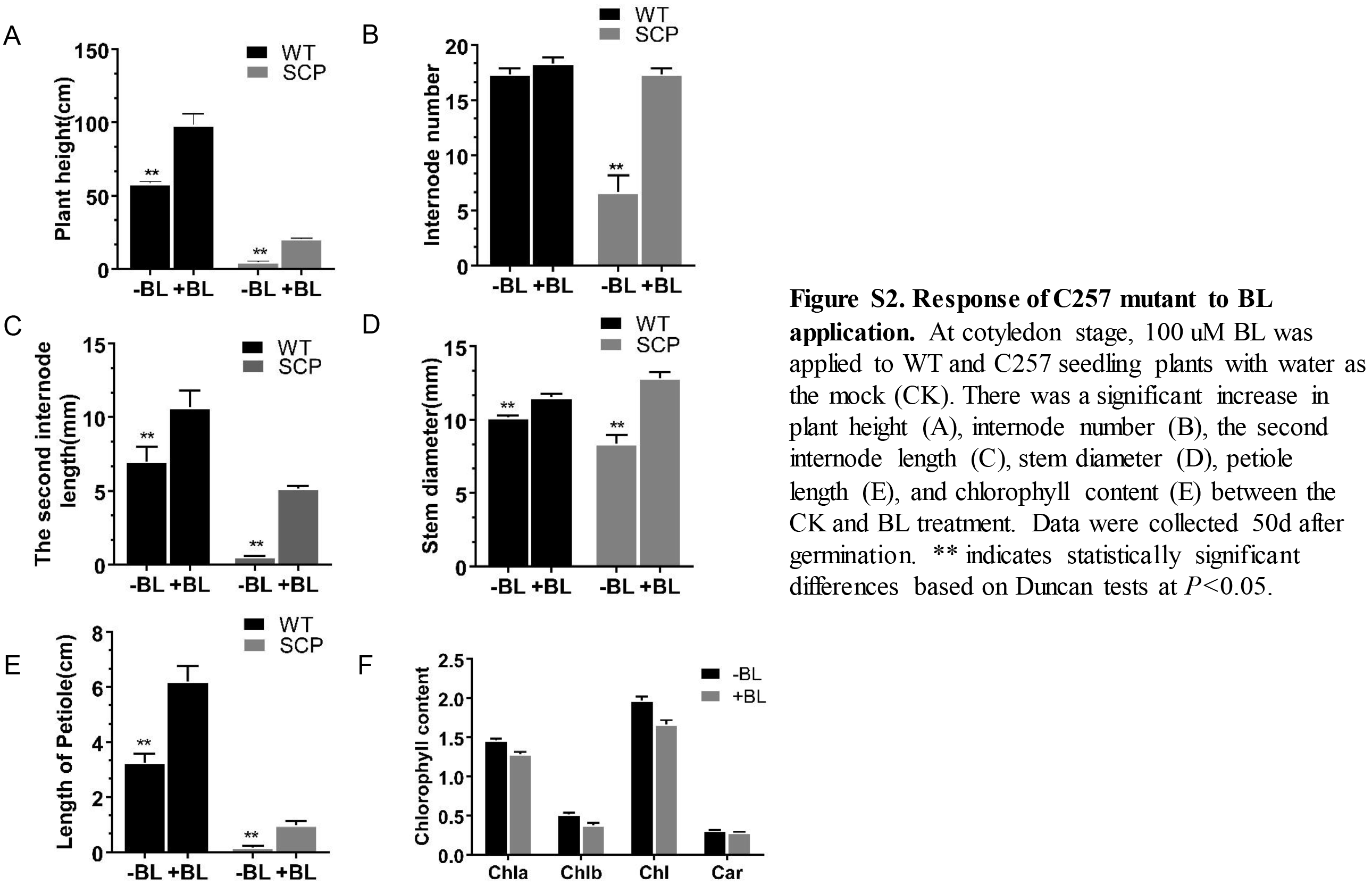

Supplement: Supplementary file 4 [file Image_2.TIF]

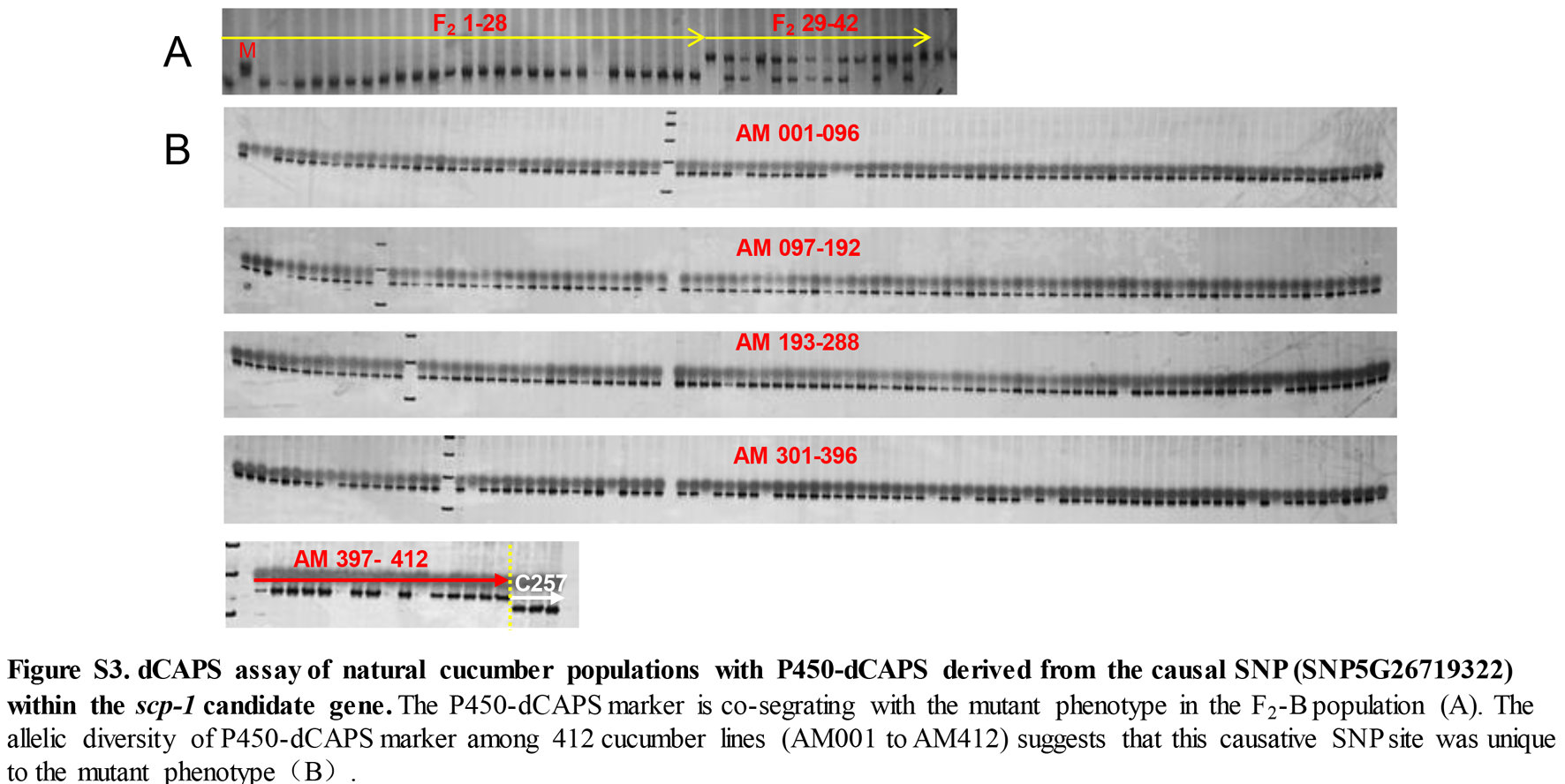

Supplement: Supplementary file 5 [file Image_3.TIF]

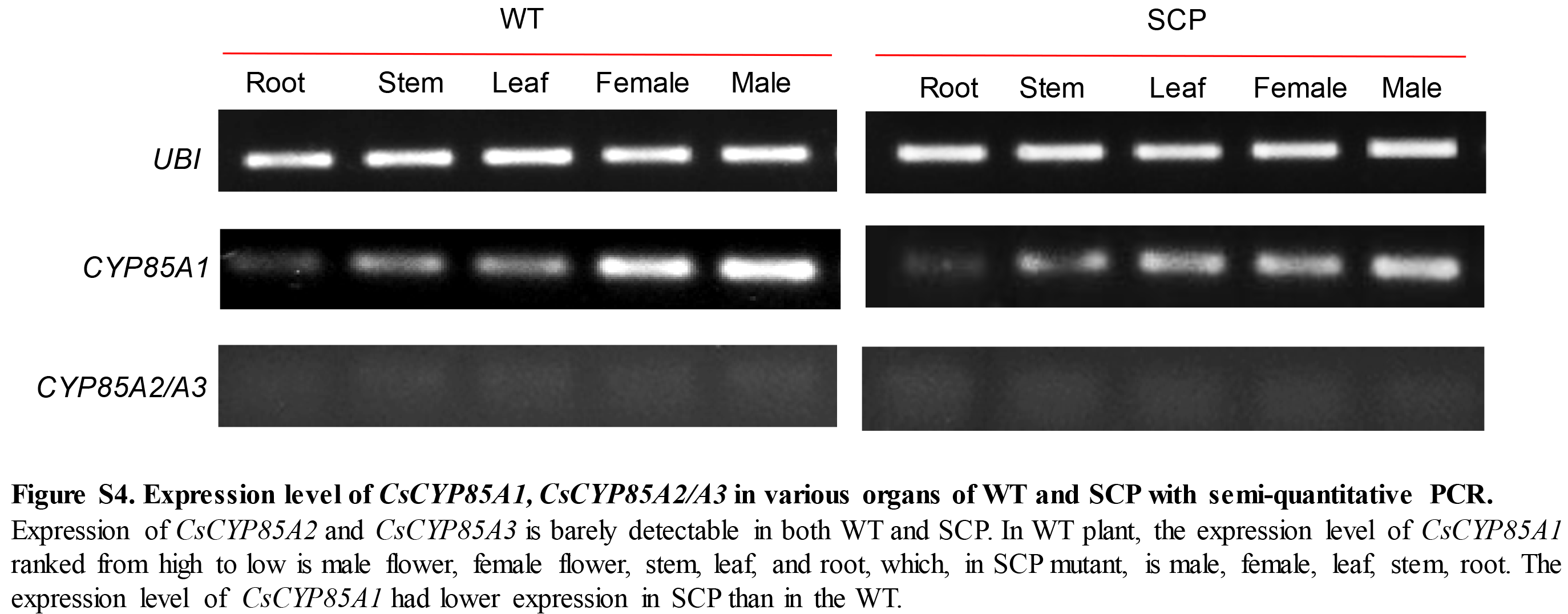

Supplement: Supplementary file 6 [file Image_4.TIF]

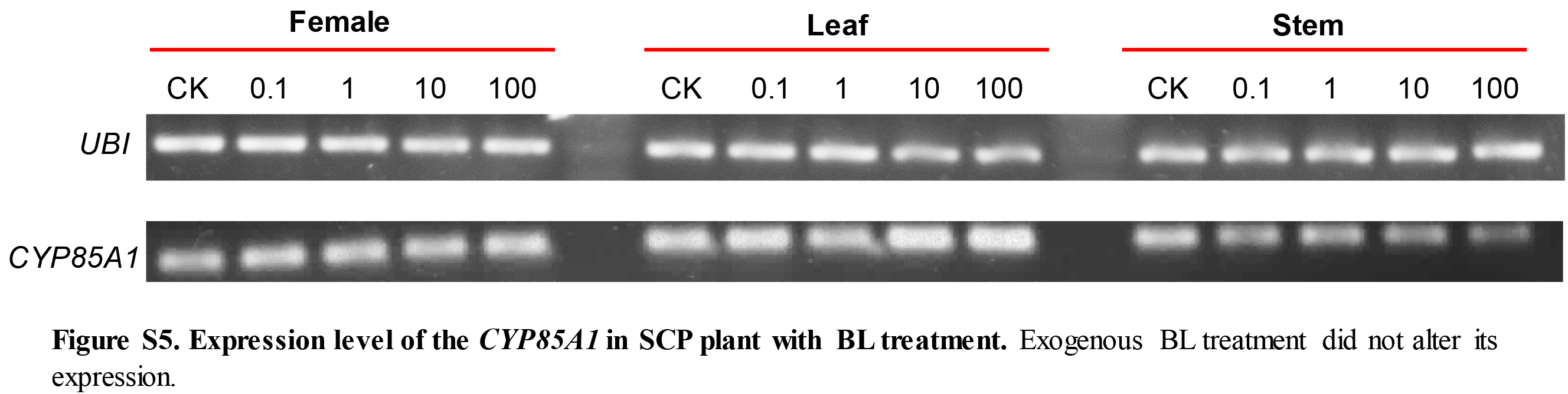

Supplement: Supplementary file 7 [file Image_5.TIF]
